# Anti-Chlamydial Th17 Responses Are Controlled by the Inducible Costimulator Partially through Phosphoinositide 3-Kinase Signaling

**DOI:** 10.1371/journal.pone.0052657

**Published:** 2012-12-20

**Authors:** Xiaoling Gao, Mathieu Gigoux, Jie Yang, Julien Leconte, Xi Yang, Woong-Kyung Suh

**Affiliations:** 1 Laboratory of Infection and Immunity, Department of Medical Microbiology, Faculty of Medicine, University of Manitoba, Winnipeg, Manitoba, Canada; 2 Immune Regulation Laboratory, Institut de recherches cliniques de Montréal, Montréal, Québec, Canada; 3 Department of Microbiology and Immunology, Faculty of Medicine, McGill University, Montréal, Québec, Canada; 4 Département de microbiologie et immunologie, Université de Montréal, Montréal, Québec, Canada; 5 Département de médecine, Université de Montréal, Montréal, Québec, Canada; Louisiana State University, United States of America

## Abstract

We previously showed that mice deficient in the Inducible Costimulator ligand (ICOSL-KO) develop more severe disease and lung pathology with delayed bacterial clearance upon respiratory infection of *Chlamydia muridarum*. Importantly, the exacerbation of disease in ICOSL-KO mice was seen despite heightened IFN-γ/Th1 responses, the major defense mechanisms against *Chlamydia*. To gain insight into the mechanism of ICOS function in this model, we presently analyzed anti-*Chlamydia* immune responses in mice lacking the entire ICOS (ICOS-KO) versus knock-in mice expressing a mutant ICOS (ICOS-Y181F) that has selectively lost the ability to activate phosphoinositide 3-kinase (PI3K). Like ICOSL-KO mice, ICOS-KO mice showed worse disease with elevated IFN-γ/Th1 responses compared to wild-type (WT) mice. ICOS-Y181F mice developed much milder disease compared to ICOS-KO mice, yet they were still not fully protected to the WT level. This partial protection in ICOS-Y181F mice could not be explained by the magnitude of IFN-γ/Th1 responses since these mice developed a similar level of IFN-γ response compared to WT mice. It was rather IL-17/Th17 responses that reflected disease severity: IL-17/Th17 response was partially impaired in ICOS-Y181F mice compared to WT, but was substantially stronger than that of ICOS-KO mice. Consistently, we found that both polarization and expansion of Th17 cells were partially impaired in ICOS-Y181F CD4 T cells, and was further reduced in ICOS-KO CD4 T cells *in vitro*. Our results indicate that once the IFN-γ/Th1 response is above a threshold level, the IL-17/Th17 response becomes a limiting factor in controlling *Chlamydia* lung infection, and that ICOS plays an important role in promoting Th17 responses in part through the activation of PI3K.

## Introduction

*Chlamydia* species are obligatory intracellular bacteria that cause reproductive defects, blindness, or pneumonia [1]. *Chlamydia trachomatis* is the most common cause of sexually transmitted diseases in humans. Safe vaccines with high efficacy have not been made available and understanding the host immune components that control *Chlamydia* infection is crucial to designing effective vaccines. Infection of mice with *Chlamydia muridarum* through pulmonary or genital route has provided relevant model systems to study the immunobiology of *Chlamydia* infection.

The main immune component that provides host protection against *Chlamydia muridarum* is CD4 T cells that express IFN-γ (Th1) [2]–[4]. Antibodies raised against *Chlamydia* provide protection after active or passive immunization [2], [5], but are dispensable for protective immunity during the primary infection [6], [7]. Recent results from *in vitro* and *in vivo* experiments suggest that IL-17 and/or CD4 T cells polarized to produce IL-17 (Th17) play important roles in the protective immunity against *Chlamydia* infections, especially in the lung [8]–[10].

The Inducible Costimulator (ICOS) is a member of the CD28 family of T cell costimulatory receptors that is expressed in activated CD4 and CD8 T cells [11]. By binding to its ligand, ICOS ligand (ICOSL), ICOS potentiates T cell proliferation and cytokine production [11]–[14]. Early studies have shown that ICOS primarily regulates Th2 and antibody responses [13]–[15]. Thus, ICOS-KO mice showed impaired Th2 responses with often elevated Th1 responses [13], [16], [17]. However, later studies have indicated that ICOS also plays important roles in Th1 and Th17 responses in multiple immunological settings [17]–[21]. We recently began to investigate the signal transduction pathways that impact on the overall function of ICOS by generating a knock-in strain of mice, termed ICOS-Y181F or ICOS-YF, in which ICOS has selectively lost the ability to activate phosphoinositide 3-kinase (PI3K) while still maintaining an intact capacity to potentiate TCR-mediated calcium mobilization [22]. Initial analysis of ICOS-YF mice revealed that ICOS utilizes PI3K signaling to promote the generation of follicular helper T cells (Tfh) that facilitate B cell growth and differentiation in the germinal center during antibody generation. Thus, ICOS-YF mice have similar defects as ICOS-KO mice in germinal center reaction, Ab class switch and affinity maturation. It has yet to be seen whether ICOS utilizes PI3K-independent signaling capacities (such as calcium mobilization) in other settings of immune responses.

We have previously shown that mice lacking the ligand for ICOS (ICOSL) have a severe defect in controlling *Chlamydia* growth in the lung, leading to drastic weight loss and lung pathology [23]. This demonstrated a critical role for the ICOS-ICOSL costimulatory axis in anti-chlamydial immunity. Contrary to the prediction that elevated susceptibility to *Chlamydia* should correlate with a weaker Th1 response, we found that the Th1 response was substantially augmented in ICOSL-KO mice throughout the course of respiratory *Chlamydia* infection [23]. These findings suggest that there are probably additional anti-*Chlamydia* immune components that are disrupted in the absence of ICOS-ICOSL costimulation.

To gain more insight into the molecular basis of ICOS function during the clearance of *Chlamydia* infection, we analysed immune responses during pulmonary infection of *Chlamydia muridarum* in ICOS-WT, ICOS-KO, and ICOS-YF mice in a *Chlamydia*-resistant C57BL/6 background. Congruent to our previous results obtained with ICOSL-KO mice, we confirmed that ICOS-KO mice experienced more severe weight loss, delayed bacterial clearance, and lung pathology with heightened Th1/IFN-γ responses during *Chlamydia* infection compared to WT mice. In contrast, we found that ICOS-YF mice were substantially better than ICOS-KO mice in controlling *Chlamydia*. Importantly, the differences in the capacity to control *Chlamydia* infection between genotypes did not correlate with the magnitude of Th1/IFN-γ responses, but rather correlated with IL-17/Th17 responses. Consistently, we revealed that the generation of Th17 cells depends on ICOS-mediated costimulation in part through the ICOS-PI3K signaling axis. Therefore, our results highlight the importance of Th17 responses in the effective control of *Chlamydia* respiratory infection in a setting of sufficient IFNγ/Th1 responses, and that ICOS-PI3K signaling plays an important role in this process.

## Results

### Contribution of ICOS-PI3K signaling to the clearance of *Chlamydia* lung infection

**Figure 1:**
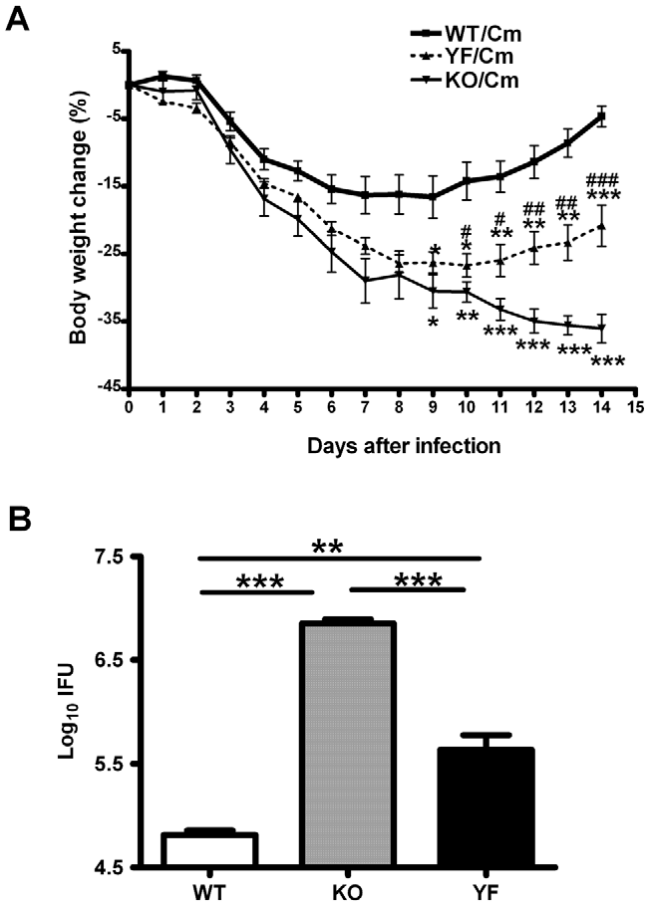
ICOS-YF mice display partially impaired ability to control *Chlamydia* infection. Three groups of mice, ICOS-WT, KO, and YF were intranasally infected with 1000 IFUs of *Chlamydia* and monitored daily for 14 days. **A** Time course of body weight change. The original body weights of the three groups were similar and the body weight changes were plotted as percentage of the original weight. Asterisks (*) on KO line indicate statistical significance of KO verse WT, while asterisks on YF line indicate statistical significance of YF vs WT. Pounds (#) on YF line indicate statistical significance of KO vs YF. **B** Bacterial burdens in the lung. Mice were euthanized at day 14 post-infection and lungs were aseptically collected and processed to assess bacterial growth *in vivo* as described in *Materials and Methods*. Data are presented as mean ± SD (7 ICOS-WT, 4 ICOS-KO, and 7 ICOS-YF mice) from one representative experiment out of three independent experiments with consistent results. * or # *p*<0.05, ** or ## *p*<0.01, *** or ### *p*<0.001.

**Figure 2:**
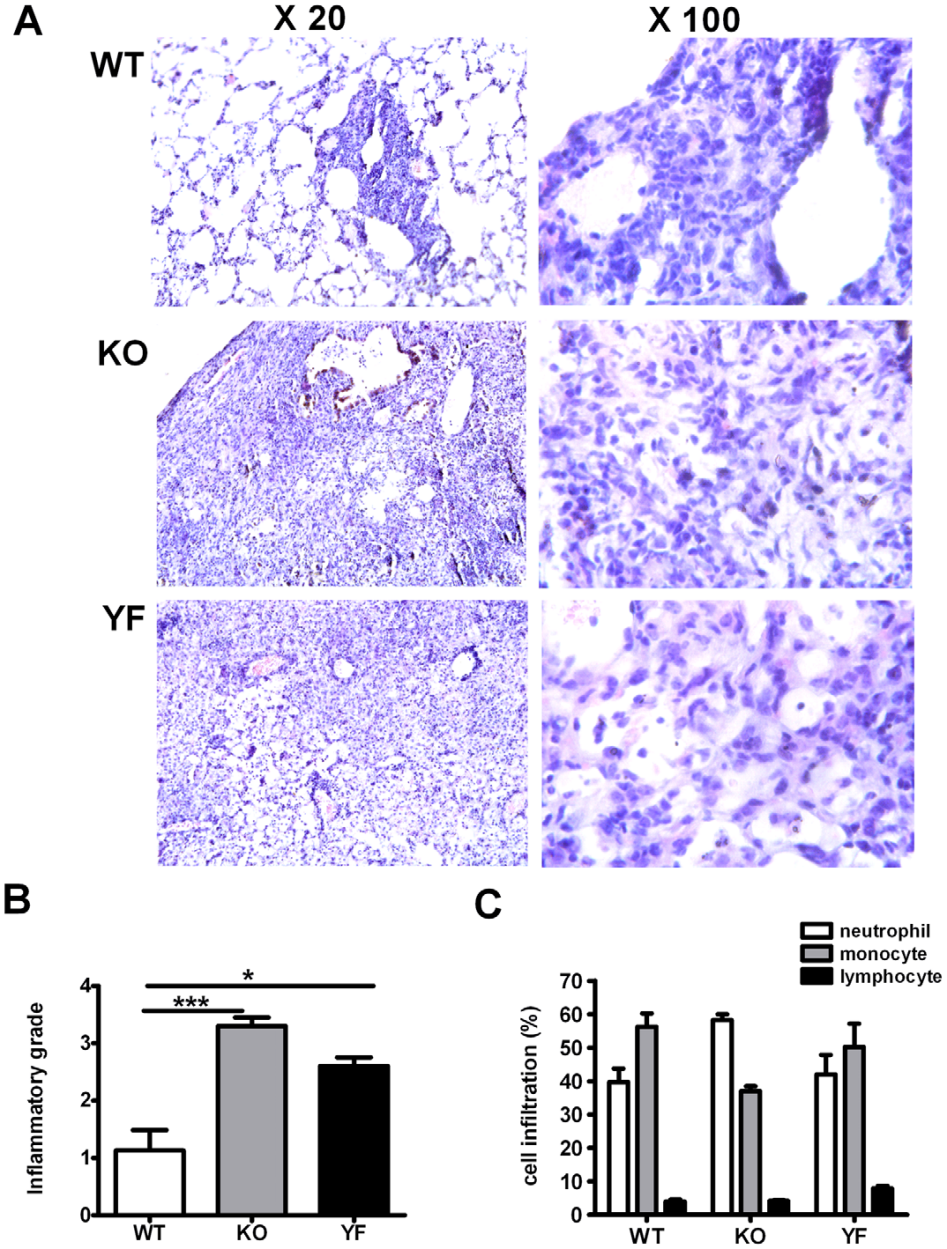
Severity of lung inflammation corresponds to the level of bacterial load. Lungs were isolated at day 14 post-infection and processed to stain with hematoxylin and eosin as described in *Materials and Methods*. **A** Representative images of the lung tissues were shown at low (20X) and high (100X) magnification. **B** and **C** Semi-quantitative analysis of lung inflammation. Slides were examined by a blinded pathologist and the inflammatory grades (**B**) and the composition of immune cell infiltrates (**C**) were analyzed as described in *Materials and Methods*. Data represent mean ± SD of 4 slides from 4 mice per group. * *p*<0.05, *** *p*<0.001.

In order to evaluate the contribution of PI3K signaling pathways in overall ICOS function in protective immunity against *Chlamydia* lung infection, we analyzed disease progression in ICOS-WT, ICOS-KO, and ICOS-YF mice in C57BL/6 background. Consistent with our previous findings made with ICOSL-KO mice, ICOS-KO mice developed more drastic weight loss compared to WT mice during the early phase of infection. Furthermore, ICOS-KO mice continued to lose weight beyond day 10 post-infection, whereas WT mice started to recover (Fig. 1A). Notably, the pattern of weight loss in ICOS-YF mice closely resembled that of ICOS-KO mice at the early phase. However, at the later phase of infection (around day 10 post-infection), ICOS-YF mice started to recover body weight similar to WT mice. This intermediate phenotype of ICOS-YF mice was reflected by the bacterial burden in the lung (14 days post-infection): it was 10-fold higher than that of WT mice but 10-fold lower than that of ICOS-KO mice (Fig. 1B). Histopathological analysis showed that WT mice nearly resolved inflammation on day 14 post-infection, exhibiting patchy peribronchial cellular infiltrates mainly composed of mononuclear cells and most area (over 80%) showed only a few perivascular infiltrates. Neutrophils represented 30–40% of infiltrating cells in the lung of WT mice. In contrast, the lung tissues of ICOS-KO mice showed much broader area of consolidation and extensive cellular infiltration with inflammatory cells, the majority of which being neutrophils (60–70%). ICOS-YF mice still had substantial lung inflammation but less infiltrating cells compared to ICOS-KO mice (Fig. 2). Collectively, these results demonstrate that the ICOS-ICOSL pathway plays a critical role in protective immunity to *Chlamydia* lung infection and that the ICOS-PI3K signaling axis substantially contributes to the overall function of ICOS in this situation.

### Altered IFN-γ responses do not correlate with clearance of *Chlamydia*

**Figure 3:**
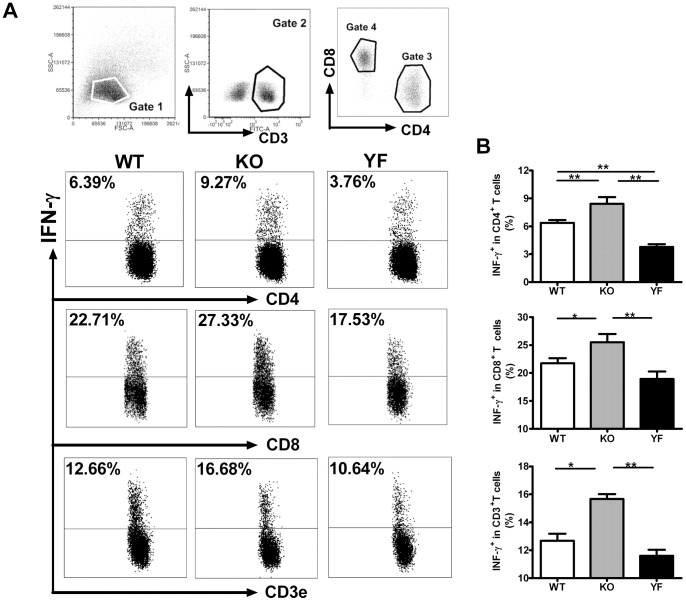
IFN-γ producing T cells in the spleen increased in ICOS-KO mice but marginally decreased in ICOS-YF mice. Splenocytes were prepared at day 14 post-infection and the IFNγ producing cells were quantified by intracellular cytokine staining as described in *Materials and Methods*. Representative flow cytometric plots (**A**) and a summary of the results (**B**) are shown. Data are presented as mean ± SD (7 ICOS-WT, 4 ICOS-KO, and 7 ICOS-YF mice) from one representative experiment out of three independent experiments with consistent results. * *p*<0.05, ** *p*<0.01, *** *p*<0.001.

**Figure 4:**
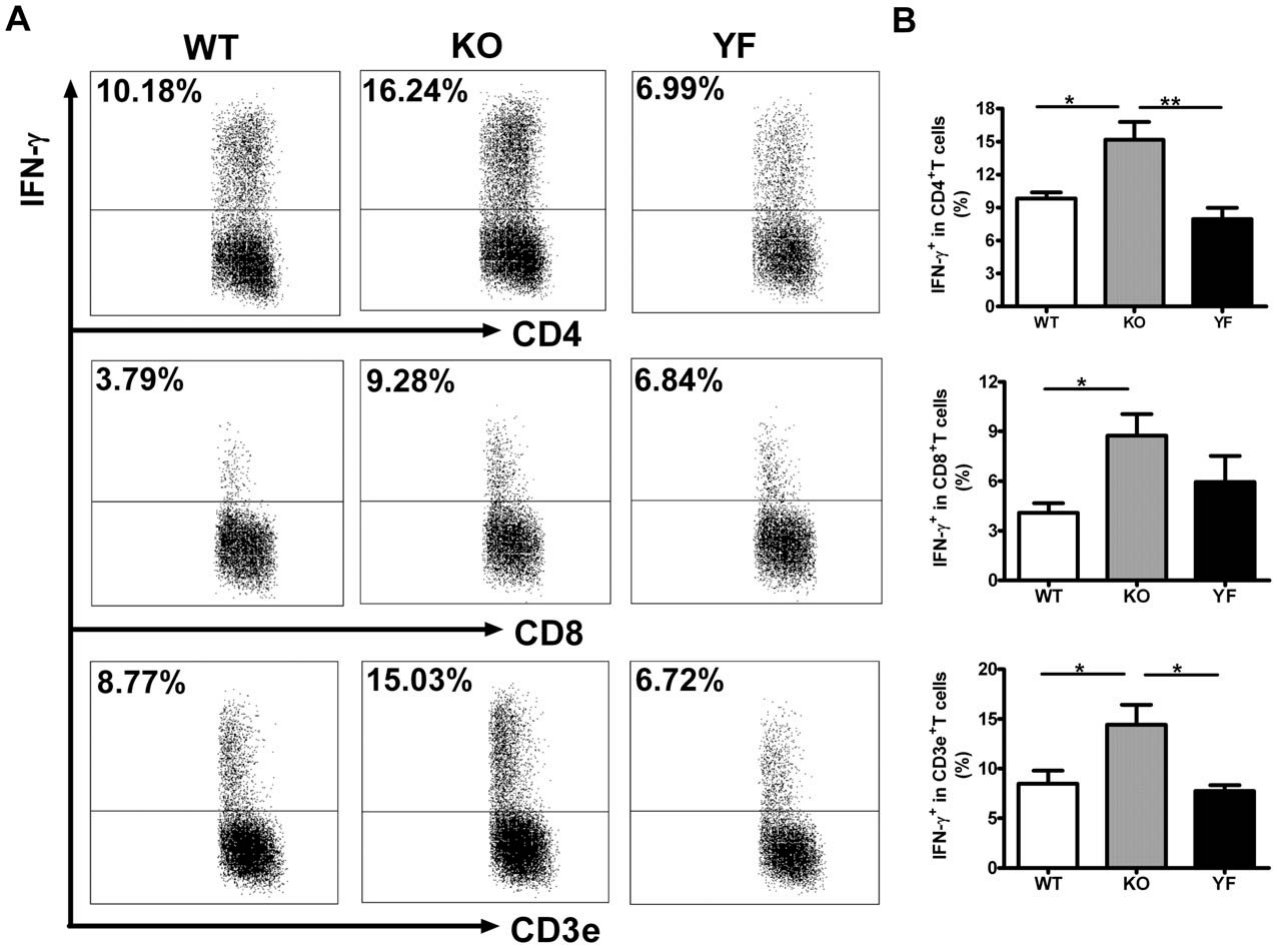
IFN-γ producing T cells in the lung increased in ICOS-KO mice but remained the same in ICOS-YF mice. Lung mononuclear cells were prepared at day 14 post-infection and the IFN-γ producing cells were quantified by intracellular cytokine staining as described in *Materials and Methods*. Representative flow cytometric plots (**A**) and a summary of the results (**B**) are shown. Data are presented as mean ± SD (7 ICOS-WT, 4 ICOS-KO, and 7 ICOS-YF mice) from one representative experiment out of three independent experiments with consistent results. * *p*<0.05, ** *p*<0.01.

**Figure 5:**
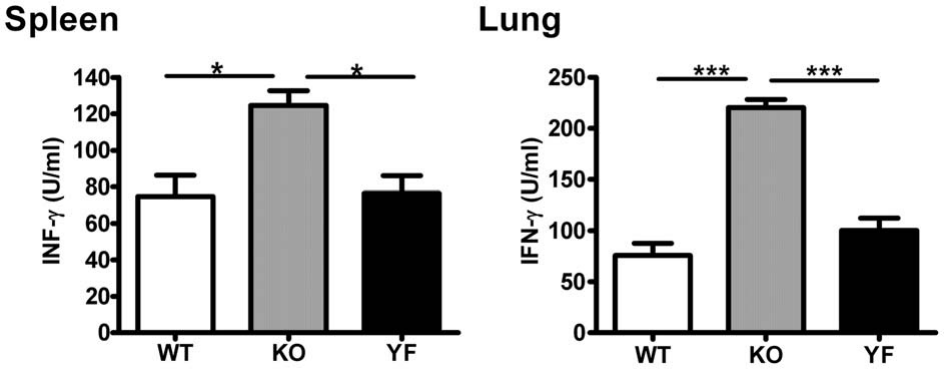
ICOS-KO cells but not ICOS-YF cells show an elevated IFN-γ production compared to WT cells upon restimulation. Single cell suspensions of splenocytes or lung mononuclear cells were prepared at day 14 post-infection and were restimulated *in vitro* as described in *Materials and Methods*. The amounts of IFN-γ in culture supernatants were measured by ELISA. Data are presented as mean ± SD (7 ICOS-WT, 4 ICOS-KO, and 7 ICOS-YF mice) from one representative experiment out of three independent experiments with consistent results. * *p*<0.05, *** *p*<0.001.

IFN-γ is considered to be the major cytokine providing anti-*Chlamydia* protection. However, we have shown that ICOSL-KO mice fail to control bacterial burden and develop more severe lung pathology although they produce much higher levels of IFN-γ, and type 1 immune responses [23]. To test the role of ICOS-PI3K signaling in type 1 responses, we analyzed IFN-γ producing CD4, CD8 T cells in the spleen (Fig. 3) and in the lung (Fig. 4) by intracellular cytokine staining. Consistent with the observation made with ICOSL-KO mice, ICOS-KO mice showed higher frequencies of IFN-γ producing T cells (both CD4 and CD8) in the spleen and lung compared to WT mice. We also measured the total amount of IFN-γ produced by total splenocytes or lung mononuclear cells after restimulation with UV-killed *Chlamydia* (Fig. 5). In keeping with the frequencies of IFN-γ producing T cells, ICOS-KO cells made 2 to 3 –fold more IFN-γ compared to WT cells. On the other hand, ICOS-YF mice showed a lower frequency of IFN-γ producing CD4 and CD8 T cells in the spleen relative to WT mice. However, the frequency of IFN-γ producing T cells in the lungs of ICOS-YF mice was not significantly different from that of WT mice (Fig. 4). Importantly, both splenocytes and lung mononuclear cells from ICOS-YF mice produced the same amount of IFN-γ as cells from WT mice upon restimulation (Fig. 5). Overall, these data show that the magnitude of IFN-γ response is augmented by elimination of ICOS, both in the frequency of IFN-γ producing T cells and the total amount of IFN-γ. However, IFN-γ response remains the same when only the PI3K signaling capacity of ICOS is selectively removed. Also, the differences in IFN-γ production do not reflect the differences in disease severity between genotypes, suggesting that immune components other than IFN-γ/Th1 should be involved in bacterial clearance.

### Disease severity is correlated with Th17 responses

**Figure 6:**
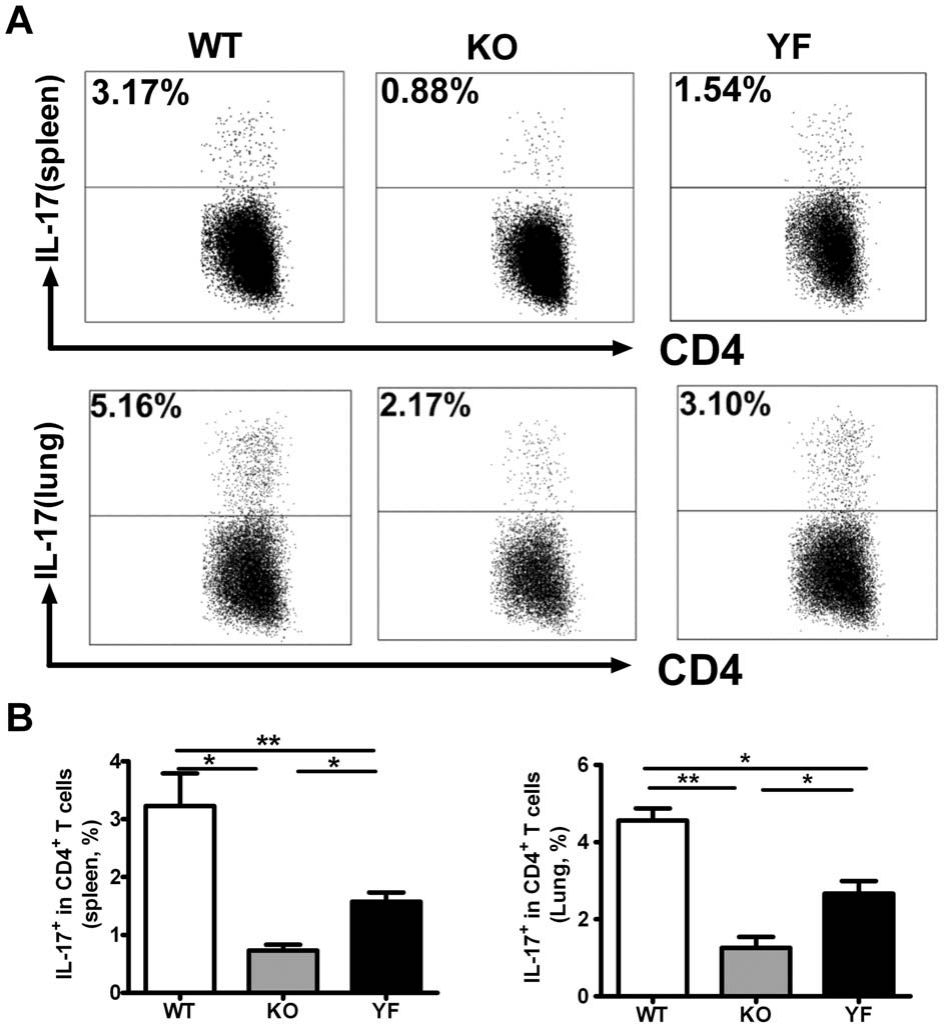
Reduced Th17 responses in ICOS-KO and ICOS-YF mice correspond to disease severity. Single cell suspensions of spleen and lung mononuclear cells of WT, ICOS-KO and ICOS-YF mice were prepared at day 14 post-infection and the IL-17 expressing CD4 T cells were detected by intracellular cytokine staining as described in *Materials and Methods*. Representative flow cytometric plots (**A**) and a summary of the results (**B**) are shown. Data are presented as mean ± SD (7 ICOS-WT, 4 ICOS-KO, and 7 ICOS-YF mice) from one representative experiment out of three independent experiments with consistent results. * *p*<0.05, ** *p*<0.01.

**Figure 7:**
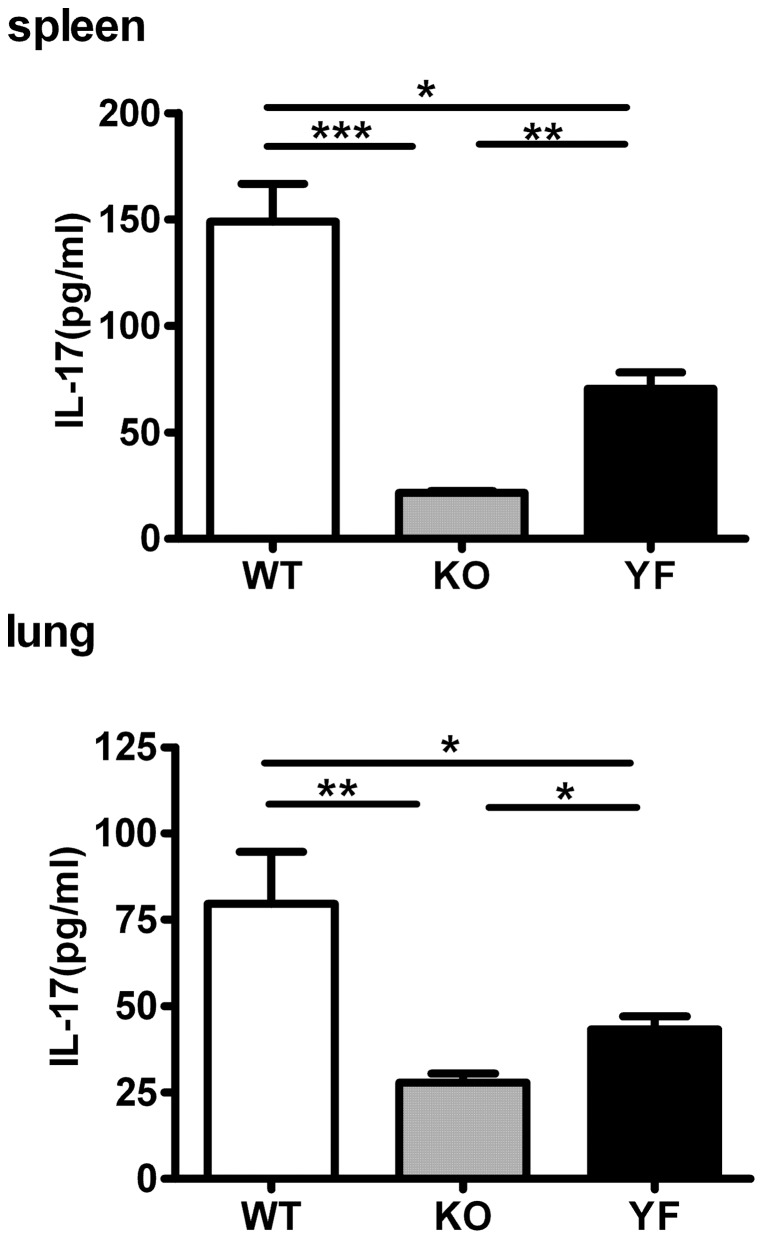
Reduced levels of IL-17 production in ICOS-KO and ICOS-YF cells upon restimulation correspond to disease severity. Single cell suspensions of splenocytes or lung mononuclear cells were prepared at day 14 post-infection and were restimulated *in vitro* as described in *Materials and Methods*. The amounts of IL-17 in culture supernatants were measured by ELISA. Data are presented as mean ± SD (7 ICOS-WT, 4 ICOS-KO, and 7 ICOS-YF mice) from one representative experiment out of three independent experiments with consistent results. * *p*<0.05, ** *p*<0.01, *** *p*<0.001.

It is becoming clear that IL-17 plays an important role in the protection against intracellular bacterial pathogens [24]–[29]. We have previously shown that ICOSL-KO mice have reduced numbers of Th17 cells during *Chlamydia* lung infection [23]. Moreover, we recently uncovered the synergistic effects of IL-17 and IFN-γ in promoting iNOS gene expression and NO production from lung epithelial cells and macrophages, thereby inhibiting the growth of *Chlamydia* in infected cells [10]. Therefore, we tested whether Th17/IL-17 responses can be a limiting factor in the protective immunity against *Chlamydia* lung infection. As shown in Fig. 6, ICOS-KO mice displayed a 3 to 4-fold reduction in the frequency of Th17 cells compared to that of WT control mice in the spleen as well as in the lung. Interestingly, ICOS-YF mice showed intermediate levels of Th17 cells relative to WT and ICOS-KO mice. The same pattern was observed when the total amount of IL-17 was measured by ELISA in the culture supernatants of *in vitro* restimulated splenocytes or lung mononuclear cells (Fig. 7). These results indicate that Th17 responses during *Chlamydia* lung infection depend on ICOS signaling, and that PI3K serves as an important downstream mediator in this process. Furthermore, the differential levels of Th17 responses are consistent with the differences in bacterial clearance and disease severity observed during *Chlamydia* infection, suggesting that Th17/IL-17 becomes a crucial immune component in the clearance of *Chlamydia* when IFN-γ is not limited.

### ICOS-PI3K signaling facilitates the generation of Th17 cells

**Figure 8:**
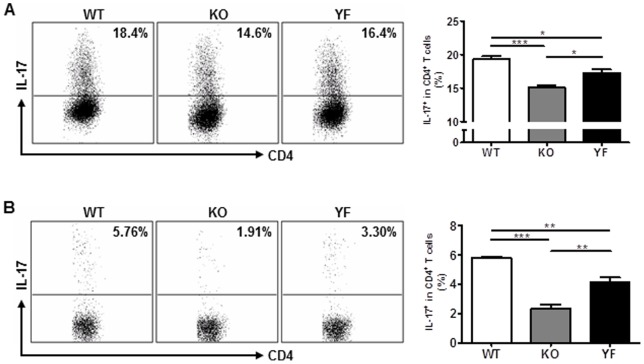
Altered Th17 responses in ICOS mutant CD4 T cells. **A** OT-II TCR transgenic CD4 T cells with ICOS-WT, KO, and YF genotype were polarized to Th17 lineage *in vitro* as described in *Materials and Methods*. The percentages of IL-17 producing cells among CD4+ CD44+ cells are depicted. **B** LN CD4 T cells purified from ICOS-WT, KO, and YF mice were stimulated in the presence of IL-23 as described in *Materials and Methods*. The percentages of IL-17 producing cells among CD4+ CD44+ cells are depicted. Data are presented as mean ± SD (4 replicates of ICOS-WT, ICOS-KO, and ICOS-YF CD4 T cell cultures) from one representative experiment out of three independent experiments with consistent results. * *p*<0.05, ** *p*<0.01, *** *p*<0.001.

Differentiation of Th17 cells is comprised of at least two phases [30]. Initially, naive CD4 T cells become polarized towards the Th17 lineage in the presence of TGF-β and IL-6. Subsequently, committed Th17 cells terminally differentiate and/or expand to form stable Th17 cells in response to IL-23 [30]. Although the steps of Th17 differentiation are difficult to separate during an immune response, they can be experimentally segregated in *in vitro* cultures. It has been shown that ICOS is required for efficient IL-17 production by *in vitro* stimulated CD4 T cells [17], as well as for IL-23-mediated expansion of Th17-committed precursor cells that exist in the pool of effector/memory CD4 T cells [19]. To understand the contribution of ICOS-PI3K signaling in Th17 generation, we first tested whether ICOS signaling affects initial Th17 polarization *in vitro*. We chose a system in which ICOS signals are delivered during T cell-APC contact using OT-II TCR transgenic CD4 T cells and peptide-loaded splenocytes as APCs. As shown in Fig. 8A, we detected small but consistent differences in the frequency of IL-17 producing cells between ICOS-WT, ICOS-KO, and ICOS-YF CD4 T cells under standard Th17 polarization conditions. Next, we analyzed the expansion of Th17 pools that were formed in the presence of intact or altered ICOS signaling. To this end, we purified CD4 T cells from lymph nodes and subsequently stimulated them *in vitro* with irradiated splenocytes supplemented with anti-CD3 antibodies and IL-23. Clearly, ICOS-KO CD4 T cells showed a ∼2.5-fold reduction in the expansion of Th17 cells compared to the WT control, whereas ICOS-YF CD4 T cells had an intermediate level of defect (Fig. 8B). Thus, ICOS plays a substantial role in the polarization and expansion of Th17 cells and PI3K is an important signaling component of ICOS signaling in this process.

### Antibody titers are affected by ICOS mutations

**Figure 9:**
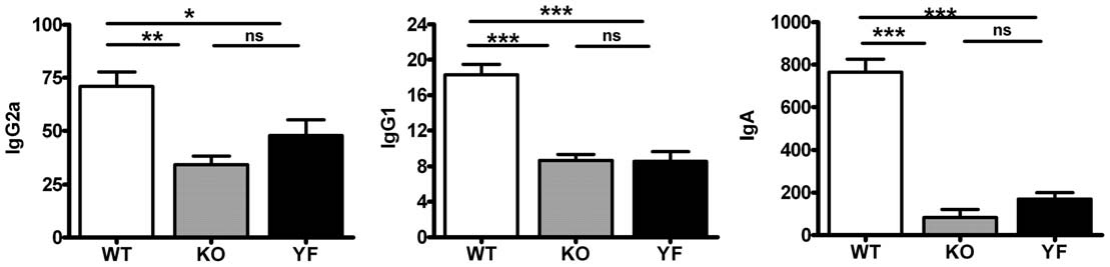
Impaired antibody responses in ICOS-KO and ICOS-YF mice. Lung homogenates were collected at day 14 post-infection and the *Chlamydia*-specific IgG2a, IgG1 and IgA titers were measured by ELISA as described in *Materials and Methods*. Data are presented as mean ± SD (7 ICOS-WT, 4 ICOS-KO, and 7 ICOS-YF mice) from one representative experiment out of three independent experiments with consistent results.* *p*<0.05, ** *p*<0.01, *** *p*<0.001.

Our previous work showed that ICOSL-KO mice had defects in the production of class-switched antibodies against *Chlamydia* [23]. Consistently, we found that ICOS-KO mice had substantially reduced anti-Chlamydial IgG2a, IgG1, and IgA titers in the lung (Fig. 9). ICOS-YF mice showed similar defects in IgG1 titer but they displayed slightly higher IgG2a and IgA levels compared to ICOS-KO mice. However, these differences were minimal in magnitude and did not provide statistically significant results. These data indicate that ICOS plays an important role in antibody production during *Chlamydia* infection and ICOS-PI3K signaling axis is heavily involved in this process. However, the overall pattern of Ab defects does not appear to explain the differences in bacterial clearance between genotypes.

## Discussion

In this study, we showed the critical importance of ICOS signaling, especially the ICOS-PI3K signaling axis, in IL-17/Th17 responses in *C. muridarum* lung infection, and the correlation of IL-17/Th17 responses with protection against the pathogen in a situation where IFN-γ production is not limited. Specifically, we found that ICOS-KO mice had a severe defect in clearing *C. muridarum* lung infection, whereas ICOS-YF mice displayed a partially impaired capacity to do so. The extent of impairment correlated with the different levels of IL-17/Th17 response in ICOS mutant mice; ICOS-YF mice showed higher IL-17/Th17 responses than ICOS-KO mice, although both of them had lower IL-17/Th17 responses than WT mice. In addition, we found that CD4 T cells from ICOS mutant mice had corresponding defects in the generation of Th17 cells. These findings provide new insight into the role of IL-17/Th17 responses in the host defense against *C. muridarum* lung infections and the mechanism of ICOS function in Th17 responses.

IFN-γ/Th1 has been shown to play a major role in protective immunity against *Chlamydia* respiratory infection [31], [32]. However, it is also known that the level of IFN-γ response does not always correlate with the level of protection [33]. In particular, we reported previously that ICOSL-KO mice suffered from higher *Chlamydia* burdens despite producing more IFN-γ [23]. The reason behind this apparent paradox has not been fully explained in the previous study. In the present study, we shed light on this issue while analyzing anti-chlamydial immune responses in ICOS mutant mice possessing different extents of signaling defects. We confirmed that ICOS-KO mice have the same phenotypes as ICOSL-KO mice following chlamydial infection, consistent with the notion that ICOSL is the sole ligand of ICOS. Moreover, we found that ICOS-YF mice, which produced higher levels of IL-17 than ICOS KO mice, had less severe disease than ICOS KO mice. Notably, both strains of mutant mice produced normal or even higher levels of IFN-γ compared to WT mice. These findings suggest that IL-17 becomes a critical factor in host defense against *Chlamydia* when IFN-γ production is sufficient or excessive. In keeping with this idea, our recent data revealed that IL-17 augments IFN-γ-mediated expression of inducible nitric oxide synthase (iNOS) in *Chlamydia*-infected lung epithelial cells and macrophages, leading to higher NO production and more efficient inhibition of intracellular bacterial growth [10]. In contrast, IL-17 on its own was unable to inhibit the intracellular growth of *Chlamydia* [9], [10]. This work also showed that neutralization of IL-17 during *Chlamydia* infection in mice reduced iNOS expression and increased the bacterial burden in the lung [10]. Combined with data in the present study, these results support a model in which IL-17 and IFN-γ work as synergistic anti-chlamydial factors, yet IL-17 can play a decisive role when IFN-γ is not limited. Of note, this scenario may be highly dependent on a specific mucosal microenvironment since the IL-17 response is dispensable in the clearance *Chlamydia muridarum* in the genital tract, presumably due to compensatory protection mechanisms provided by macrophages [34]. It is also important to note that our proposed model is based on an acute lung pathology that reflects bacterial persistence and thus may not be applicable to a genital tract infection of *Chlamydia* in which upper reproductive tract sequelae do not correlate with bacterial burdens [35].

Our *in vitro* data demonstrated that ICOS-PI3K signaling is required for the optimal polarization and expansion of Th17 cells. These results are consistent with the findings linking ICOS and PI3K activity to IL-17 production or Th17 differentiation [17], [19], [36], [37]. However, the underlying molecular mechanisms remain to be elucidated.

Since we found that ICOS-YF mice develop only partial defects in Th17 response compared to that of ICOS-KO mice during *Chlamydia* infection, this suggests that the residual signaling capacities of ICOS-YF T cells (e.g., intracellular calcium mobilization) contribute to the generation/expansion of Th17 cells. Therefore, further study of the PI3K-dependent and -independent ICOS signaling pathways related to IL-17/Th17 responses in *Chlamydia* infection is important to better understand the mechanism of ICOS-ICOSL signaling in modulating host defense.

We showed previously that ICOS-YF mice had as severe defects as ICOS-KO mice in the generation of class-switched IgG Abs when immunized with protein antigens with alum [22]. Consistently, data in this study showed that ICOS-YF mice had similar defects in Ab responses against Chlamydial lung infection compared to ICOS-KO mice. Overall, these results suggest that the PI3K-dependent signaling pathways of ICOS play a bigger role in supporting follicular helper T cell responses compared to Th17 responses.

Taken together, our results suggest that ICOS provides a balancing function that regulates Th1 and Th17 responses to control *Chlamydia* respiratory infection. In addition to its function to preferentially promote Th2 over Th1 responses, ICOS can facilitate Th17 response partially through PI3K. Thus, lack of ICOS results in heightened Th1/IFN-γ responses along with a severe reduction in Th17 responses leading to a serious defect in bacterial clearance in ICOS-KO mice. In contrast, in ICOS-YF mice, abrogation of ICOS-mediated PI3K pathways partially reduces Th17 with unaltered IFN-γ responses causing a partial defect in bacterial control. These results indicate that an optimal combination of Th1 and Th17 responses is important for controlling *Chlamydia* lung infection and warrant further studies on whether this synergy operates in other settings of intracellular bacterial infection.

## Materials and Methods

### Animals

ICOS-KO and ICOS-YF mouse strains have been previously described [13], [22]. Both strains of mice were backcrossed for ten generations onto C57BL/6 background and wild type (WT) littermates from ICOS-YF breeding or inbred C57BL/6 mice were used as WT control with similar results. Female mice with ICOS-WT, ICOS-YF, and ICOS-KO at 7–10 weeks of age were used for experiments. TCR transgenic OT-II mice and C57BL/6 mice were purchased from Jackson Laboratory. OT-II transgenic mice were bred with ICOS-YF or ICOS-KO mice to generate OT-II+ ICOS mutant mice. For infection experiments, mice were bred in the specific pathogen-free environment at the IRCM and transferred to the animal facility at University of Manitoba. This study was carried out in strict accordance with the recommendations in the Canadian Council on Animal Care (CCAC) Guidelines and according to animal use protocols approved by the Animal Care Committee of IRCM and the Protocol Management and Review Committee of University of Manitoba.

### Preparation of *Chlamydia*

*Chlamydia muridarum* was propagated, purified and quantified as previously described [38]. Briefly, *C. muridarium* was grown in HeLa-229 cells in MEM medium containing 10% fetal bovine serum (FBS) and 2 mM glutamine. Elementary bodies (EBs) were purified by discontinuous density gradient centrifugation. Titers of EBs were determined by measuring inclusion forming units (IFU) after immunostaining and aliquots of the EB stock were stored at −80°C.

### Infection of *Chlamydia* and quantification of bacterial load

Mice were sedated with isoflurane and infected intranasally with 1×10^3^ IFUs of *C. muridarum* in 40 μl sucrose-phosphate-glutamic acid (SPG) buffer. Mouse body weight was monitored daily. Fourteen days after infection, mice were euthanized and the lungs were aseptically isolated and homogenized using a cell grinder in SPG buffer. The tissue homogenates were centrifuged and supernatant were stored at −80°C until being tested. The bacterial load in *C. muradium*-infected lungs was assessed as previously described [39]. Briefly, 100 μl of serially diluted organ tissue supernatants was inoculated onto HeLa-229 monolayers for 2 hours before MEM medium were supplied to each well. After 48 h-incubation, cells were fixed with methanol and stained with *Chlamydia*-specific mAb and HRP-conjugated secondary antibodies. The bacterial inclusions were visualized using chromogenic substrate 4-chloro-1-naphthol (Sigma) and enumerated under the microscope.

### Analysis of lung pathology

Lung sections were evaluated by a pathologist in a blinded manner after staining with hematoxylin and eosin. The degree of lung inflammation was scored using a semi-quantitative grading system: 0, normal; 1, mild and limited inflammation, granuloma formation, cellular infiltration in less than 25% of area, no obvious infiltration into adjacent alveolar septae or air space; 2, mild interstitial pneumonitis, diffused cellular infiltration in some area (25–50%), septal congestion, interstitial edema; 3, inflammatory cell infiltration into perivascular, peribronchiolar, alveolar septae, and air space (50%–75% of area); 4, over 75% of area of lung filled with infiltrating cells. For profiling of immune cell infiltration, five microscopic high-power fields were randomly selected per slide. Neutrophils, monocytes, and lymphocytes were differentially enumerated until the total count reached 200 cells per field and the average numbers were calculated from all the sampled fields.

### Flow cytometry

Splenocytes and lung mononuclear cells were aseptically prepared from mice at d14 post-infection. Single cell suspensions were stimulated with PMA (50 ng/ml)/ionomycin (1 μg/ml) (Sigma) for 6 hours in the presence of 20 μg/ml brefeldin A (Sigma). The cells were washed with FACS buffer (Dulbecco's PBS, 2% heat-inactivated FBS, 0.05% sodium azide) twice and incubated with anti-mouse CD16/32 (Fcblock; eBioscience) for 20 min on ice. Cells were subsequently stained for surface markers with anti-CD3e, anti-CD4, anti-CD8, anti-CD44, and isotype control Abs for 30 min on ice. The cells were fixed and permeabilized with intracellular cytokine staining reagents (eBioscience) and subsequently stained with anti-IFN-γ-APC, IL-17-APC, or corresponding isotype control Abs (eBioscience). The raw data were collected using LSR II flow cytometer (BD Biosciences), and the data were analyzed using FACS Express software (De Novo Software).

### ELISA

*C. muridarum*-specific serum IgA, IgG1, and IgG2a were determined by ELISA as previously described [40]. Results are expressed as relative titers calculated from dilution folds that gave OD value of 0.5 at 405 nm. For quantification of cytokines, mice were sacrificed on day 14 post-infection. Single cell suspensions of splenocytes (7.5×10^5^ cells/well) and lung mononuclear cells (5×10^5^ cells/well) were cultured with UV-killed *C. muridarum* for 72 h in 96-well plates. The culture supernatants were collected and measured for IFN-γ and IL-17 by ELISA using Abs from eBioscience.

### Th17 culture

For *in vitro* polarization of Th17 cells, splenic CD4 T cells were purified (95%) from OT-II TCR transgenic mice with different ICOS genotypes using mouse CD4+ T cell enrichment kit (STEMCELL Technologies). Purified CD4 T cells were stimulated with irradiated C57BL/6 splenocytes loaded with ovalbumin 323–339 peptides (3 μM) for 4 days in the presence of human TGFβ1 (3 ng/ml; R&D Systems) and IL-6 (20 ng/ml; eBioscience) in RPMI media supplemented with 10% FBS and antibiotics. For Th17 expansion experiments, CD4 T cells were purified from a pool of cervical, axillary, inguinal, and mesenteric lymph nodes isolated from ICOS-WT or ICOS mutant mice using mouse CD4+ T cell enrichment kit (STEMCELL Technologies). The purified CD4 T cells were incubated with irradiated autologous splenocytes in the presence of anti-CD3 (1 μg/ml; eBioscience) and IL-23 (20 ng/ml; R&D Systems) for 4 days. At the end of the culture, IL-17 producing cells among CD4+ CD44+ T cells were assessed by intracellular cytokine staining.

### Statistical analysis

Statistical analysis of the data was performed using ANOVA (GraphPad Prism software, version 4), values of p<0.05 were considered significant. Data are presented as mean ± SD unless otherwise indicated. The presented data were collected from 3∼7 ICOS-WT, 3∼4 ICOS-KO, and 3∼7 ICOS-YF mice. All the experiments were repeated two to three times with similar results.
